# Clinical characteristics associated with recurrent viral RNA positivity in patients within two weeks after recovering from the first SARS-CoV-2 infection

**DOI:** 10.17305/bb.2023.9661

**Published:** 2024-02-01

**Authors:** Xi Cao, Yongli Xie, Chunlei Zhou, Hong Mu

**Affiliations:** 1 Department of Clinical Laboratory, Tianjin First Central Hospital, Tianjin, China; 2 Department of Clinical Laboratory, Tianjin Stomatological Hospital, Tianjin, China; 3 Department of Tianjin Key Laboratory of Oral and Maxillofacial Function Reconstruction, Tianjin Stomatological Hospital, Tianjin, China

**Keywords:** Severe acute respiratory syndrome coronavirus 2 (SARS-CoV-2), recurrent viral RNA positivity (RP), interleukin 6 (IL-6), neutrophil to lymphocyte ratio (NLR)

## Abstract

Many studies have shown that recovered coronavirus disease 2019 (COVID-19) patients frequently exhibit recurrent viral RNA positivity (RP) for the severe acute respiratory syndrome coronavirus 2 (SARS-CoV-2). Our study aimed to summarize the clinical characteristics of these patients and explore potential reasons for RP occurrence. We divided 439 participants into four groups based on the severity of illness prior to the COVID-19 recovery and age: mild-child group, moderate-child group, mild-adult group, and moderate-adult group. Laboratory data were collected and statistically analyzed using the SPSS software, version 24.0. Significant differences were observed in age, alanine aminotransferase (ALT), aspartate aminotransferase (AST), C-reactive protein (CRP), interleukin 6 (IL-6), and neutrophil to lymphocyte ratio (NLR) levels between the mild-adult group and the moderate-adult group (*P* < 0.05). Additionally, AST levels differed significantly between the mild-child group and the moderate-child group (*P* < 0.05). The proportion of RP patients within the four groups varied from 7.95% to 26.13% within a 2-week period. Logistic regression analysis revealed that younger age and moderate symptoms were risk factors for RP in children, while the presence of comorbidities (such as chronic heart, lung, liver, and kidney diseases), elevated IL-6 levels, and NLR were risk factors for RP in adults. We constructed two predictive models containing these relevant parameters, and the results of the receiver operating characteristic (ROC) curves indicated strong predictive utility. Our findings suggest that younger children with more severe symptoms, as well as adult patients with elevated levels of IL-6 and NLR and underlying diseases, are at higher risk of RP occurrence.

## Introduction

Since the global outbreak of the coronavirus disease 2019 (COVID-19), reverse transcription-polymerase chain reaction (RT-PCR) has been greatly useful in the detection of the severe acute respiratory syndrome coronavirus 2 (SARS-CoV-2) due to its specificity and simplicity as a qualitative assay in many health sectors. Additionally, RT-PCR offers sufficient sensitivity to be highly useful in diagnosing early infections. According to the World Health Organization (WHO), patients infected with SARS-CoV-2 can be discharged from hospitals after demonstrating significant improvement in their symptoms, along with the confirmation of two negative RT-PCR tests within 24 h [1]. However, a critical issue has recently emerged regarding recurrent viral RNA positivity (RP) in some recovered COVID-19 patients [2]. Various studies have reported that the proportion of RP cases among discharged COVID-19 patients varied from 2.4% to 69.2%, the age of RP patients spans from 0 to 91 years, and the recurrence can last from 1 to 38 days after discharge [3, 4]. Nevertheless, there remains a limited understanding of the characteristics of RP patients. Further exploration of the risk factors associated with RP occurrence is needed.

The objective of this article is to estimate the incidence of RP and to investigate the characteristics and risk factors associated with RP in both children and adult patients who have recovered from COVID-19. In doing so, we hope to provide valuable insights for clinical prevention and control strategies.

## Materials and methods

In this study, 439 recovered COVID-19 patients were investigated at the Tianjin First Central Hospital, Tianjin, China, from March 2022 to May 2023. The participants were categorized into four groups based on age and the severity of illness prior to the COVID-19 recovery: mild-child group, moderate-child group, mild-adult group, and moderate-adult group. Data, including symptoms recorded at admission, comorbidities, laboratory results in biochemistry and hematology, and nucleic acid results within two weeks, were obtained from the COVID-19 rehabilitation ward at the Tianjin First Central Hospital.

Following the WHO guidelines [1], we retrospectively enrolled 439 recovered COVID-19 patients, all of whom had achieved clinical recovery and had received two consecutive negative RT-PCR tests for SARS-CoV-2, separated by at least 24 h. Daily RT-PCR tests were performed during the patients’ recovery period. Total nucleic acids were extracted using the RNA virus kits (Zybio, China), and subsequently analyzed through RT-PCR. To improve the virus detection rate, we selected kits from three different manufacturers for cross-comparison. Each patient’s sample was tested using commercial SARS-CoV-2 kits provided by three different manufacturers (Zybio, Zhi Jiang, and Bo Jie, all based in China). The cycle threshold (Ct) value for each sample was calculated according to the manufacturing instructions, and the threshold Ct value for a positive result was set at 40, as per the China Technical Guidelines for Laboratory Testing for COVID-19.

The mild and moderate diagnoses were determined based on guidelines from the National Health Commission of China. Patients aged 14 and under were included in the child groups, while those aged 20 and over were placed in the adult groups. To minimize the incidence of false-negative results, nasopharyngeal swabs were performed on all patients during the two-week observation period.

### Ethical statement

All patients signed an informed consent form to participate in this study. This study was reviewed and approved by the Medical Ethics Committee of Tianjin First Central Hospital (Ethics Committee archiving No. 2022N052KY) and conformed to the principles outlined in the Declaration of Helsinki.

### Statistical analysis

Statistical analysis was performed using the SPSS 24.0 for Windows software (SPSS, Chicago, IL, USA). Group variables were represented by numbers (%), normally distributed continuous variables were represented by means ± standard deviations (SDs), and non-normally distributed continuous variables were represented by medians. Either analysis of variance (ANOVA) or the Mann–Whitney *U* test was used for comparisons between the two groups. Risk factors, including continuous variables and adjusted outcome variables for RP, were evaluated through logistic regression analysis. Statistical significance was defined as a *P* value < 0.05 or a 95% confidence interval (CI) not including 1.0. The performance of prognostic models was evaluated using the receiver operating characteristic (ROC) curve analysis. An area under the ROC curve (AUROC) value close to 1.0 indicates high diagnostic accuracy. According to the principle that the maximum of the Youden index corresponds to the best sensitivity and specificity, a *P* value < 0.05 was considered statistically significant, and a *P* value < 0.001 was considered highly statistically significant.

## Results

### Study cohort characteristics

In this retrospective study, 439 patients who had recovered from COVID-19 were categorized into four cohorts based on age and the severity of illness prior to recovery. The mild-child group consisted of 88 mild COVID-19 child subjects, the moderate-child group included 39 moderate COVID-19 child subjects, the mild-adult group had 113 mild COVID-19 adult subjects, and the moderate-adult group comprised 199 moderate COVID-19 adult subjects. The gender distribution was as follows: approximately 47.73% of the mild-child group, 56.41% of the moderate-child group, 46.90% of the mild-adult group, and 41.71% of the moderate-adult group were male. The mean ages of the four groups were 8.74 ± 3.20, 8.85 ± 3.12, 40.02 ± 13.91, and 50.30 ± 15.31 years, respectively. The clinical characteristics pertaining to biochemistry and hematology are presented in Table 1.

**Table 1:** Baseline clinical and laboratory characteristics of the COVID-19 recovered patients

**Variables**	**Mild-child group**	**Moderate-child group**	**Mild-adult group**	**Moderate-adult group**
Cases, *n*	88	39	113	199
Male, *n* (%)	42 (47.73)	22 (56.41)	53 (46.90)	83 (41.71)
Age (years)	8.74 ± 3.20	8.85 ± 3.12	40.02 ± 13.91	50.30 ± 15.31
GLU (mmol/L)	4.36 ± 0.37	4.24 ± 0.31	5.16 ± 1.43	5.47 ± 1.35
AST (IU/L)	27.98 ± 6.27	32.81 ± 9.09	31.90 ± 20.28	44.54 ± 60.63
ALT (IU/L)	17.09 ± 10.16	21.18 ± 18.89	40.64 ± 37.39	67.48 ± 81.07
CRE (µmol/L)	38.99 ± 8.45	38.88 ± 5.11	60.93 ± 14.70	60.37 ± 14.01
URE (mmol/L)	4.03 ± 1.19	3.88 ± 0.97	4.03 ± 1.23	4.31 ± 1.33
IgG (g/L)	155.13 ± 72.59	165.97 ± 51.02	187.61 ± 61.16	199.12 ± 60.01
IgM (g/L)	0.77 ± 1.05	0.78 ± 0.86	1.33 ± 2.94	3.93 ± 28.70
CRP (mg/L)	1.38 ± 5.53	0.67 ± 0.64	1.54 ± 2.12	2.92 ± 7.44
TG (mmol/L)	1.10 ± 0.50	1.22 ± 0.49	1.96 ± 1.21	2.13 ± 1.36
ALB (g/L)	47.20 ± 2.47	47.46 ± 2.63	47.33 ± 3.13	46.62 ± 2.79
TC (mmol/L)	4.42 ± 0.75	4.69 ± 0.95	5.30 ± 1.00	5.11 ± 1.12
TP (g/L)	72.24 ± 3.40	71.76 ± 3.53	74.17 ± 4.55	73.53 ± 4.56
WBC (×10^9^/L)	7.51 ± 2.15	6.89 ± 2.43	7.39 ± 1.83	7.66 ± 1.95
RBC (×10^12^/L)	4.81 ± 0.28	4.79 ± 0.45	4.90 ± 0.61	4.84 ± 0.48
HGB (g/L)	136.55 ± 7.94	138.39 ± 16.13	145.97 ± 19.96	145.12 ± 15.58
PLT (×10^9^/L)	339.46 ± 68.80	319.36 ± 80.80	275.82 ± 62.18	273.36 ± 70.82
IL-6 (U/mL)	2.28 ± 3.34	1.68 ± 0.51	2.15 ± 1.62	3.31 ± 5.68
MONO (%)	5.04 ± 1.03	4.85 ± 0.94	4.96 ± 1.27	5.01 ± 1.42
LYM (%)	38.50 ± 8.65	41.03 ± 4.03	28.96 ± 7.21	27.06 ± 7.54
BASO (%)	0.36 ± 0.19	0.40 ± 0.11	0.39 ± 0.23	0.37 ± 0.23
EO (%)	1.98 ± 1.74	2.34 ± 1.04	1.60 ± 1.26	1.63 ± 1.42
NEUT (%)	54.12 ± 9.81	50.72 ± 4.42	64.09 ± 7.75	65.94 ± 8.40
NLR	1.56 ± 0.76	1.25 ± 0.20	2.43 ± 0.98	2.81 ± 1.60

Data are presented as *n* (%), and mean ± standard deviation. The 439 patients who had recovered from COVID-19 were categorized into four cohorts based on age and the severity of illness prior to recovery. The mild and moderate diagnoses were determined based on guidelines from the National Health Commission of China. Patients aged 14 and under were included in the child groups, while those aged 20 and over were placed in the adult groups. COVID-19: Coronavirus disease 2019; GLU: Glucose; AST: Aspartate aminotransferase; ALT: Alanine aminotransferase; CRE: Creatinine; URE: Urea; IgG: Immunoglobulin G; IgM: Immunoglobulin M; CRP: C-reactive protein; TG: Triglycerides; ALB: Albumin; TC: Total cholesterol; TP: Total protein; WBC: White blood cells; RBC: Red blood cells; HGB: Hemoglobin; PLT: Platelets; IL-6: Interleukin 6; MONO: Monocytes; LYM: Lymphocytes; BASO: Basophils; EO: Eosinophils; NEUT: Neutrophils; NLR: Neutrophil to lymphocyte ratio.

### Statistical comparison of clinical parameters among recovered patients

We conducted a statistical comparison of the clinical laboratory parameters between the child groups and the adult groups, specifically contrasting the mild-child group with the moderate-child group and the mild-adult group with the moderate-adult group. Significant differences were observed in age, aspartate aminotransferase (AST), alanine aminotransferase (ALT), C-reactive protein (CRP), interleukin 6 ( IL-6), and neutrophil to lymphocyte ratio (NLR) between the mild-adult group and the moderate-adult group. Additionally, a statistically significant difference in AST was also noted between the mild-child group and the moderate-child group (*P* < 0.01) (Table 2). The AST levels were significantly higher in the moderate-child group compared to the mild-child group. Similarly, age and the levels of AST, ALT, CRP, IL-6, and NLR in the moderate-adult group were notably higher compared to those in the mild-adult group. These laboratory markers are indicative of disease severity, and the results suggested that the inflammatory markers in patients (whether adults or children) with moderate symptoms were more pronounced than those in patients with mild symptoms (Table 2).

**Table 2:** Statistical comparison of clinical parameters between the different groups of COVID-19-recovered patients

**Variables**	**Mild-child group vs moderate-child group**	**Mild-adult group vs moderate-adult group**
Age (years)	0.857	< 0.001***
Sex	0.371	0.375
GLU (mmol/L)	0.112	0.079
AST (IU/L)	0.001**	0.03*
ALT (IU/L)	0.118	0.001**
CRE (µmol/L)	0.939	0.739
URE (mmol/L)	0.505	0.074
IgG (g/L)	0.428	0.111
IgM (g/L)	0.632	0.345
CRP (mg/L)	0.332	0.045*
TG (mmol/L)	0.298	0.312
ALB (g/L)	0.666	0.061
TC (mmol/L)	0.149	0.185
TP (g/L)	0.552	0.277
WBC (x 10^9^/L)	0.213	0.261
RBC (x 10^12^/L)	0.751	0.356
HGB (g/L)	0.322	0.705
PLT (x 10^9^/L)	0.216	0.777
IL-6 (U/mL)	0.265	0.02*
MONO (%)	0.391	0.779
LYM (%)	0.153	0.047*
BASO (%)	0.366	0.388
EO (%)	0.381	0.842
NEUT (%)	0.098	0.079
NLR	0.051	0.033*

The numbers represent *P* values. The patients who had recovered from COVID-19 were categorized into four cohorts based on age and the severity of illness prior to recovery. The mild and moderate diagnoses were determined based on guidelines from the National Health Commission of China. Patients aged 14 and under were included in the child groups, while those aged 20 and over were placed in the adult groups. The results of the statistical comparison of clinical parameters between the two child and the two adult groups are shown in the table. *P < 0.05; **P < 0.01; ***P < 0.001. COVID-19: Coronavirus disease 2019; GLU: Glucose; AST: Aspartate aminotransferase; ALT: Alanine aminotransferase; CRE: Creatinine; URE: Urea; IgG: Immunoglobulin G; IgM: Immunoglobulin M; CRP: C-reactive protein; TG: Triglycerides; ALB: Albumin; TC: Total cholesterol; TP: Total protein; WBC: White blood cells; RBC: Red blood cells; HGB: Hemoglobin; PLT: Platelets; IL-6: Interleukin 6; MONO: Monocytes; LYM: Lymphocytes; BASO: Basophils; EO: Eosinophils; NEUT: Neutrophils; NLR: Neutrophil to lymphocyte ratio.

### Recurrent viral RNA positivity in recovered patients

In the four groups, RP within two weeks was observed in seven patients from the mild-child group (7.95%), seven patients from the moderate-child group (17.95%), 16 patients from the mild-adult group (14.15%), and 52 patients from the moderate-adult group (26.13%) (Table 3). Excluding children, approximately 21.24% of patients in the mild-adult group and 43.72% of patients in the moderate-adult group had various comorbidities. Details on the comorbidities among adult patients are presented in Table 3. The proportion of patients with chronic heart or lung disease was slightly lower, while the percentage of patients with diabetes, liver, or kidney disease was higher.

**Table 3:** Comorbidities and the proportion of RP patients in the four groups

**Variables**	**Mild-child group**	**Moderate-child group**	**Mild-adult group**	**Moderate-adult group**
Patients	88	39	113	199
Comorbidities	None	None	24 (21.24%)	87 (43.72%)
Chronic heart/lung disease	None	None	5 (4.42%)	20 (10.05%)
Diabetes	None	None	9 (7.96%)	31 (15.58%)
Chronic liver/kidney disease	None	None	8 (7.08%)	28 (14.07%)
Others	None	None	2 (1.78%)	8 (4.02%)
RP patients	7 (7.95%)	7 (17.95%)	16 (14.15%)	52 (26.13%)

Data are presented as *n* and *n* (%). The patients who had recovered from COVID-19 were categorized into four cohorts based on age and the severity of illness prior to recovery. The mild and moderate diagnoses were determined based on guidelines from the National Health Commission of China. Patients aged 14 and under were included in the child groups, while those aged 20 and over were placed in the adult groups. RP: Recurrent viral RNA positivity; COVID-19: Coronavirus disease 2019.

### Construction and assessment of predicting models for recurrent viral RNA positivity based on multiple parameters

Furthermore, we investigated the RP cases and compared their clinical laboratory parameters with those of other patients. Interestingly, the trends were similar to the previously mentioned results, although not identical. Among a total of 127 children patients, there were significant differences in age, severity of illness, and IL-6 levels between the RP cases and the others (*P* < 0.05) (Table S1). The IL-6 levels and severity of illness in RP cases were higher, while age was lower (Table S2). Furthermore, we found significant differences in age, severity of illness, CRP, NLR, IL-6, and the presence of comorbidities between the RP cases and other patients among the 312 adult patients (*P* < 0.05) (Table S1). All parameters in adult RP cases were significantly higher (Table S2).

**Figure 1.**
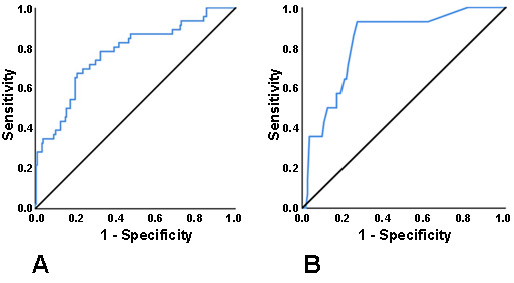
**Two multivariate logistic regression models constructed to evaluate the predictive abilities of significant parameters for RP.** (A) ROC curve constructed to predict the probability of RP cases in 312 adult patients, based on their comorbidities, IL-6 values, and NLR values. The AUROC was 0.771, and the cutoff value was set at 0.30, yielding a sensitivity of 71.4% and a specificity of 78.7%; (B) ROC curve constructed to predict the probability of RP cases in 127 child patients, based on age and severity of illness. The AUROC was 0.829, and the cutoff value was set at 0.079, yielding a sensitivity of 92.9% and a specificity of 73.2%. RP: Recurrent viral RNA positivity; ROC: Receiver operating characteristic; IL-6: Interleukin 6; NLR: Neutrophil to lymphocyte ratio; AUROC: Area under the ROC curve.

We have then used logistic regression analysis to evaluate the risk posed by these significant parameters in predicting RP among both children and adults. The main factors influencing the RP occurrence revealed three significant parameters in children and six significant parameters in adults (according to the logistic regression outcomes, predictors and protectors of RP were identified, where a 95% CI > 1 indicated a risk of the event, whereas a 95% CI < 1 indicated protection). Our results showed that age acted as a protective factor against RP in children (Table 4), while the severity of illness served as a risk factor. In adults, comorbidities, IL-6, and NLR were identified as risk factors for RP (Table 5), with comorbidities being the most strongly associated. We constructed two multivariate logistic regression models to test their predicting abilities for RP. Model A aimed to predict the probability of RP in 312 adult patients and included comorbidities, IL-6, and NLR. The AUROC was 0.771 (Figure 1A), and the cutoff value was set at 0.30, yielding a sensitivity of 71.4% and a specificity of 78.7%. Model B included two significant variables: age and severity of illness. With a predicted probability of RP in 127 child patients, the AUROC was 0.829 (Figure 1B). The cutoff value was set at 0.079, yielding a sensitivity of 92.9% and a specificity of 73.2%.

**Table 4:** Logistic regression analysis of prognostic factors for RP in children

**Variables**	**Beta coefficient**	**Standard error**	**Wald**	**OR**	**95% CI**	***P* value**
Age	−0.314	0.097	10.426	0.730	0.604 – 0.884	0.001**
IL-6	0.078	0.092	0.726	1.081	0.903 – 1.294	0.394
Severity of illness	1.227	0.642	3.656	3.410	0.970 – 11.990	0.049*

Results of the logistic regression analysis conducted to evaluate how these significant parameters predicted RP in children. *P < 0.05; **P < 0.01. RP: Recurrent viral RNA positivity; IL-6: Interleukin 6; CI: Confidence interval.

**Table 5:** Logistic regression analysis of prognostic factors for RP in adults

**Variables**	**Beta coefficient**	**Standard error**	**Wald**	**OR**	**95% CI**	***P* value**
Age	−0.015	0.013	1.289	0.985	0.959 – 1.011	0.256
Comorbidities	1.862	0.403	21.316	6.438	2.920 – 14.192	< 0.001***
Severity of illness	0.066	0.396	0.028	1.068	0.492 – 2.319	0.868
CRP	0.020	0.029	0.486	1.020	0.964 – 1.080	0.485
IL-6	0.128	0.061	4.395	1.336	1.008 – 1.480	0.036*
NLR	0.190	0.112	3.906	1.309	1.010 – 1.505	0.048*

Results of the logistic regression analysis conducted to evaluate how these significant parameters predicted RP in adults. *P < 0.05; **P < 0.01; ***P < 0.001. RP: Recurrent viral RNA positivity; CRP: C-reactive protein; IL-6: Interleukin 6; NLR: Neutrophil to lymphocyte ratio.

## Discussion

The RP among recovered COVID-19 patients is a known phenomenon, but the factors contributing to its occurrence have not been fully investigated. This study evaluated the clinical characteristics of recovered COVID-19 patients, focusing on those who exhibited RP. Our finding revealed that patients in the moderate-child group had higher levels of AST compared to those in the mild-child group. Similarly, age and the levels of AST, ALT, CRP, IL-6, and NLR were also higher in the moderate-adult group compared to those in the mild-adult group. The incidence of RP among the four groups of recovered COVID-19 patients ranged from 7.95% to 26.13%. Logistic regression analysis indicated that young children with moderate symptoms and adults with comorbidities, elevated IL-6, and NLR levels were at a higher risk of experiencing RP within two weeks.

Endothelial cells express angiotensin-converting enzyme 2 (ACE2) on their surface, which also serves as the primary receptor for SARS-CoV-2. ACE2 mediates the infection of endothelial cells, leading to endothelial activation and damage [5]. Due to the abundant expression of ACE2, COVID-19 can induce tissue injury and immune dysregulation, resulting in highly elevated levels of inflammatory cytokines, such as IL-6 and CRP [6], as well as increased NLR [7]. Our study found that the CRP, IL-6, and NLR levels were significantly higher in the moderate-adult group compared to the mild-adult group, which can be attributed to the heightened activity of the inflammatory cytokine storm in moderate-adult cases. Previous research has shown that many COVID-19 patients experience liver function abnormalities related to the severity of their illness, primarily marked by an increase in AST and ALT levels [8]. Our finding aligns with this, revealing that AST levels were significantly higher in the moderate-child group compared to the mild-child group, and both AST and ALT levels were significantly higher in the moderate-adult group compared to the mild-adult group (*P* < 0.05).

In our study, we observed differences in the incidence of RP between child and adult groups, as well as between mild and moderate severity groups. Specifically, the incidence of RP was higher in moderate groups compared to mild groups for both children (17.95% vs 7.95%) and adults (26.13% vs 14.15%). Additionally, within both mild and moderate severity categories, adults exhibited a higher incidence of RP than children (14.15% vs 7.95% for mild groups; 26.13% vs 17.95% for moderate groups). We utilized the logistic regression analysis to identify risk factors associated with RP occurrence in these groups. Interestingly, age acted as a protective factor in children (OR ═ 0.678; *P* < 0.05), and moderate symptoms emerged as a risk factor. However, age did not show statistical significance as a risk factor among adults. The most significant risk factor for adults was the presence of comorbidities (OR ═ 6.438; *P* < 0.001). Previous research has indicated that age may be a host-dependent factor influencing the occurrence of RP [9]. Wu et al. [10] reported a lower percentage of elderly people among patients with RP, which contradicts our findings. Our results demonstrated a higher rate of RP in adult patients compared to child patients (14.15% vs 7.95% for mild groups; 26.13% vs 17.95% for moderate groups), highlighting the complexity of age as a factor in RP occurrence.

In our study, the children group had no underlying comorbidities, which may partially explain the lower incidence of RP. Older children with mild symptoms appeared to achieve viral clearance earlier and more quickly. Their immune systems also seemed better equipped to defend against COVID-19 upon a second exposure. In contrast, adults were more likely to experience RP than children, which could be associated with the presence of underlying comorbidities, such as chronic heart disease or diabetes, which were more frequently observed among RP adults [11]. The immune system’s functional activity may be suppressed in adults with such comorbidities, thereby increasing their susceptibility to RP [12], as a decline in immunity can easily contribute to a resurgence of the body’s viral load and subsequent disease relapse [13]. IL-6 and NLR were identified as risk factors for RP, potentially due to their role in escalating inflammation. IL-6 serves as a proinflammatory cytokine capable of activating the intracellular Janus kinase (Jak)/signal transducer and activator of transcription (STAT) cascade and perpetuating inflammation through a STAT3-mediated positive feedback loop in non-immune cells [14]. Elevated NLR levels suggest an intensified inflammatory response and compromised immune function [15]. To synthesize these risk factors for predicting RP, we constructed two multivariate logistic regression models. The results showed that the AUROC for both models exceeded 0.75 in predicting RP, whether for adult or child patients. We propose that age, severity of illness, comorbidities, IL-6, and NLR could serve as effective indicators for predicting RP within two weeks in COVID-19-recovered patients.

This study has several limitations. First, it is a single-center retrospective study with a relatively small sample size, leading to unequal patient numbers across the groups, particularly a small number of children patients. Second, the study did not include a quantitative analysis of viral RNA and neutralizing antibodies, nor did it dynamically assess viral characteristics. Third, the applicability and clinical utility of our findings may be limited by the small sample size. Therefore, further validation is needed to determine their relevance to other populations.

## Conclusion

We examined the alterations in the clinical characteristics of recovered COVID-19 patients and identified the significant parameters predictive of RP. Our findings suggest that younger children with more severe symptoms, as well as adult patients with elevated levels of IL-6 and NLR and underlying diseases, are at higher risk of RP occurrence.

## Supplemental data

**Table S1:** Differences in clinical characteristics between the RP patients and the non-RP patients

**Variables**	**RP children vs non-RP children**	**RP adults vs non-RP adults**
Age (years)	< 0.001***	< 0.001***
Comorbidities	–	< 0.001***
Sex	0.975	0.316
Severity of illness	0.049*	0.014*
GLU (mmol/L)	0.168	0.071
AST (IU/L)	0.128	0.639
ALT (IU/L)	0.410	0.333
CRE (µmol/L)	0.331	0.897
URE (mmol/L)	0.402	0.103
IgG (g/L)	0.059	0.530
IgM (g/L)	0.194	0.531
CRP (mg/L)	0.530	< 0.001***
TG (mmol/L)	0.377	0.828
ALB (g/L)	0.269	0.110
TC (mmol/L)	0.550	0.470
TP (g/L)	0.880	0.496
WBC (×10^9^/L)	0.456	0.310
RBC (×10^12^/L)	0.534	0.284
HGB (g/L)	0.553	0.532
PLT (×10^9^/L)	0.276	0.799
IL-6 (U/mL)	0.047*	0.029*
MONO (%)	0.054	0.159
LYM (%)	0.332	0.007**
BASO (%)	0.351	0.456
EO (%)	0.902	0.108
NEUT (%)	0.120	< 0.001***
NLR	0.261	0.013*

The numbers in the table represent *P* values. *P < 0.05; **P < 0.01; ***P < 0.001. RP: Recurrent viral RNA positivity; GLU: Glucose; AST: Aspartate aminotransferase; ALT: Alanine aminotransferase; CRE: Creatinine; URE: Urea; IgG: Immunoglobulin G; IgM: Immunoglobulin M; CRP: C-reactive protein; TG: Triglycerides; ALB: Albumin; TC: Total cholesterol; TP: Total protein; WBC: White blood cells; RBC: Red blood cells; HGB: Hemoglobin; PLT: Platelets; IL-6: Interleukin 6; MONO: Monocytes; LYM: Lymphocytes; BASO: Basophils; EO: Eosinophils; NEUT: Neutrophils; NLR: Neutrophil to lymphocyte ratio.

**Table S2:** Clinical and laboratory characteristics of RP patients and non-RP patients

**Variables**	**Non-RP children**	**RP children**	**Non-RP adults**	**RP adults**
Cases, *n*	113	14	244	68
Comorbidities, *n* (%)	–	–	62 (25.41)	49 (72.06)
Male, *n* (%)	54 (47.79)	7 (50)	111 (45.49)	28 (41.17)
Age (years)	10.04 ± 0.24	8.67 ± 1.33	44.81 ± 14.60	52.69 ± 17.36
GLU (mmol/L)	4.37 ± 0.40	4.44 ± 0.10	5.27 ± 1.38	5.68 ± 1.39
AST (IU/L)	27.66 ± 0.92	31.61 ± 0.67	40.65 ± 55.18	37.39 ± 25.03
ALT (IU/L)	18.85 ± 1.72	18.29 ± 0.21	59.73 ± 73.68	50.41 ± 52.13
CRE (µmol/L)	40.62 ± 6.86	38.32 ± 2.38	60.62 ± 13.90	60.37 ± 15.53
URE (mmol/L)	3.70 ± 0.12	4.35 ± 0.40	4.09 ± 1.16	4.63 ± 1.67
IgG (g/L)	177.53 ± 35.40	156.27 ± 36.63	196.19 ± 56.69	190.91 ± 73.15
IgM (g/L)	0.73 ± 0.12	1.66 ± 0.80	3.45 ± 2.09	1.44 ± 3.04
CRP (mg/L)	1.28 ± 0.67	1.41 ± 0.11	1.67 ± 4.01	5.16 ± 10.29
TG (mmol/L)	1.55 ± 0.06	1.03 ± 0.11	2.05 ± 1.25	2.10 ± 1.51
ALB (g/L)	47.42 ± 0.30	45.90 ± 1.99	47.12 ± 2.98	45.89 ± 2.55
TC (mmol/L)	4.52 ± 0.99	4.92 ± 0.35	5.16 ± 1.05	5.29 ± 1.17
TP (g/L)	72.43 ± 0.40	73.49 ± 2.68	73.88 ± 4.39	73.37 ± 5.27
WBC (×10^9^/L)	7.34 ± 0.28	6.16 ± 0.74	7.49 ± 1.88	7.81 ± 1.88
RBC (×10^12^/L)	4.82 ± 0.03	4.90 ± 0.19	4.88 ± 0.54	4.78 ± 0.50
HGB (g/L)	139.10 ± 1.40	142.33 ± 7.69	145.78 ± 18.09	144.00 ± 14.06
PLT (×10^9^/L)	342.29 ± 8.74	312.33 ± 33.86	273.82 ± 68.87	276.63 ± 61.07
IL-6 (U/mL)	1.85 ± 0.21	2.50 ± 0.05	2.23 ± 1.80	5.30 ± 9.07
MONO (%)	4.94 ± 0.11	5.00 ± 0.62	5.08 ± 1.41	4.60 ± 1.08
LYM (%)	38.87 ± 0.90	39.23 ± 1.25	28.41 ± 7.37	25.13 ± 7.31
BASO (%)	0.38 ± 0.02	0.40 ± 0.06	0.37 ± 0.23	0.42 ± 0.21
EO (%)	2.05 ± 1.00	1.90 ± 0.15	1.59 ± 1.30	1.75 ± 1.59
NEUT (%)	53.64 ± 0.99	51.07 ± 2.69	64.56 ± 8.05	68.10 ± 8.21
NLR	1.85 ± 0.21	1.50 ± 0.01	2.06 ± 1.34	3.13 ± 1.58

Data are presented as *n* (%), and mean ± standard deviation. RP: Recurrent viral RNA positivity; GLU: Glucose; AST: Aspartate aminotransferase; ALT: Alanine aminotransferase; CRE: Creatinine; URE: Urea; IgG: Immunoglobulin G; IgM: Immunoglobulin M; CRP: C-reactive protein; TG: Triglycerides; ALB: Albumin; TC: Total cholesterol; TP: Total protein; WBC: White blood cells; RBC: Red blood cells; HGB: Hemoglobin; PLT: Platelets; IL-6: Interleukin 6; MONO: Monocytes; LYM: Lymphocytes; BASO: Basophils; EO: Eosinophils; NEUT: Neutrophils; NLR: Neutrophil to lymphocyte ratio.
